# Endothelial Dysfunction in COVID-19: A Unifying Mechanism and a Potential Therapeutic Target

**DOI:** 10.3390/biomedicines10040812

**Published:** 2022-03-30

**Authors:** Pasquale Ambrosino, Ilenia Lorenza Calcaterra, Marco Mosella, Roberto Formisano, Silvestro Ennio D’Anna, Tiziana Bachetti, Giuseppina Marcuccio, Brurya Galloway, Francesco Paolo Mancini, Antimo Papa, Andrea Motta, Matteo Nicola Dario Di Minno, Mauro Maniscalco

**Affiliations:** 1 Istituti Clinici Scientifici Maugeri IRCCS, Cardiac Rehabilitation Unit of Telese Terme Institute, 82037 Telese Terme, Italy; roberto.formisano@icsmaugeri.it (R.F.); mancini@unisannio.it (F.P.M.); antimo.papa@icsmaugeri.it (A.P.); 2 Department of Clinical Medicine and Surgery, Federico II University, 80131 Naples, Italy; ileniacalcaterra@hotmail.it; 3 Istituti Clinici Scientifici Maugeri IRCCS, Pulmonary Rehabilitation Unit of Telese Terme Institute, 82037 Telese Terme, Italy; marco.mosella@icsmaugeri.it (M.M.); silvestro.danna@icsmaugeri.it (S.E.D.); 4 Istituti Clinici Scientifici Maugeri IRCCS, Scientific Direction, 27100 Pavia, Italy; tiziana.bachetti@icsmaugeri.it; 5 Università della Campania Luigi Vanvitelli, 81100 Caserta, Italy; giuseppina_marcuccio@hotmail.it (G.M.); brurya29@gmail.com (B.G.); 6 Department of Science and Technology, University of Sannio, 82100 Benevento, Italy; 7 Institute of Biomolecular Chemistry, National Research Council, 80078 Pozzuoli, Italy; andrea.motta@icb.cnr.it; 8 Department of Translational Medical Sciences, Federico II University, 80131 Naples, Italy; mnd.diminno@gmail.com

**Keywords:** COVID-19, endothelial function, chronic obstructive pulmonary disease, occupational medicine, heart failure, chronic disease, arginine, rehabilitation, exercise, outcome

## Abstract

The novel severe acute respiratory syndrome coronavirus 2 (SARS-CoV-2) generated a worldwide emergency, until the declaration of the pandemic in March 2020. SARS-CoV-2 could be responsible for coronavirus disease 2019 (COVID-19), which goes from a flu-like illness to a potentially fatal condition that needs intensive care. Furthermore, the persistence of functional disability and long-term cardiovascular sequelae in COVID-19 survivors suggests that convalescent patients may suffer from post-acute COVID-19 syndrome, requiring long-term care and personalized rehabilitation. However, the pathophysiology of acute and post-acute manifestations of COVID-19 is still under study, as a better comprehension of these mechanisms would ensure more effective personalized therapies. To date, mounting evidence suggests a crucial endothelial contribution to the clinical manifestations of COVID-19, as endothelial cells appear to be a direct or indirect preferential target of the virus. Thus, the dysregulation of many of the homeostatic pathways of the endothelium has emerged as a hallmark of severity in COVID-19. The aim of this review is to summarize the pathophysiology of endothelial dysfunction in COVID-19, with a focus on personalized pharmacological and rehabilitation strategies targeting endothelial dysfunction as an attractive therapeutic option in this clinical setting.

## 1. Introduction

In December 2019, a novel single-stranded RNA virus, called severe acute respiratory syndrome coronavirus 2 (SARS-CoV-2), was first identified [1]. SARS-CoV-2, which can cause coronavirus disease 2019 (COVID-19), features a wide spectrum of clinical manifestations [1]. Among them, the worst clinical picture is characterized by the development of severe pneumonia, which may rapidly progress to acute respiratory distress syndrome (ARDS) and multiple organ failure (MOF) [2]. In addition, the persistence of multiple disabilities and long-term cardiovascular (CV) sequelae in COVID-19 survivors suggests that convalescent patients may suffer from post-acute COVID-19 syndrome, re-quiring long-term care and personalized rehabilitation approaches [3]. However, the pathophysiology of acute and post-acute manifestations of COVID-19 is still under investigation, as a better interpretation of these mechanisms would ensure more effective personalized therapies.

To date, growing evidence supports the key role of endothelial dysfunction in the pathogenesis of COVID-19 and in determining its severity [4]. Data in recent studies have demonstrated that severe pulmonary manifestations in COVID-19 patients are not only due to ARDS, but also to macro- and microvascular involvement, with vascular endothelial injury and subsequent dysfunction [5]. Vascular damage is probably related both to the direct cytopathic effect of the virus on endothelial cells (ECs) and to the high levels of cytokines and other inflammatory markers, inducing systemic endotheliitis, platelet activation, leucocyte adhesion, and reduced nitric oxide (NO) bioavailability [6,7]. Overall, it is evident that in COVID-19 the pathological process is not limited to the lungs, and the systemic inflammatory process is responsible for an imbalance between the prothrombotic and anticoagulant properties of the endothelium, leading to arterial and venous thrombosis [8]. Indeed, patients with severe COVID-19 frequently suffer from pulmonary and systemic vascular complications, including pulmonary embolism, deep vein thrombosis, and major CV events [9,10]. Consequently, the European Society of Cardiology (ESC) recommended clinical assessment of endothelial function in the follow-up of all convalescent COVID-19 patients to prevent long-term CV outcomes [4]. Several methods have been proposed to clinically evaluate endothelial function in humans, among which the most used is flow-mediated dilation (FMD) [11]. FMD is a non-invasive and cost-effective approach, accepted as a valid substitute indicator of subclinical atherosclerosis and coronary artery endothelial function [12]. In addition, FMD is an independent predictor of CV events [13], thus providing additional prognostic data along with conventional CV risk factors. Applying the ESC recommendations [4], several studies have begun to evaluate FMD in convalescent COVID-19 patients, substantially confirming the presence of a dysfunctional endothelium even months after disease onset [7,14]. In line with this evidence, an increased risk of incident CV disease has recently been reported during the convalescence phase, spanning several categories, including stroke, ischemic heart disease, heart failure, and thromboembolic disease [15].

As clinical evidence indicates a crucial endothelial contribution to the clinical manifestations of COVID-19, the aim of this review is to summarize the pathophysiology of endothelial dysfunction in this clinical setting, with a focus on personalized pharmacological and rehabilitation strategies targeting endothelial dysfunction as an attractive therapeutic option.

## 2. Endothelial Cell Homeostasis

The endothelium is considered a real organ, with its own defined structure capable of guaranteeing vascular homeostasis through several functions [16]. Under physiological conditions, ECs are able to respond to a number of hemodynamic and humoral stimuli by producing a wide range of mediators regulating vascular tone, cellular adhesion, coagulation, smooth muscle cell proliferation, and vessel wall inflammation [16]. However, despite all of these being defense mechanisms, these functions might become dysregulated under certain circumstances [17].

To guarantee vascular homeostasis, the endothelium first needs to maintain its intact structure. There are several molecules involved in this process and the main one is vascular endothelial-cadherin (VE-cadherin, also known as CD144), which is a component of endothelial cell-to-cell adherent junctions and a promoter of an optimal organization of ECs cytoskeleton [18]. Moreover, since the endothelium plays a crucial role in controlling immune response, it regulates leucocyte migration into extravascular spaces, defending against infections and promoting tissue repair [19]. ECs show on their surface a number of adhesion molecules (e.g., E-selectin, P-selectin), whose concentration increases in response to proinflammatory cytokines, such as interleukin (IL)-1β, IL-6, and tumor necrosis factor-α (TNF-α). Then, the binding of the leucocytes is reinforced through other adhesion molecules, including intercellular adhesion molecule-1 (ICAM-1, also known as CD54), vascular cell adhesion molecule-1 (VCAM-1, also known as CD106), and integrins [20].

Another key function that the endothelium has is the prevention of thrombosis and the activation of the coagulation cascade, which is a very complex process that involves many factors, among which the most important are platelets and ECs themselves [16]. In fact, several mechanisms can provoke endothelial activation and dysfunction through the loss of ECs structural integrity, leading to the exposure of subendothelial thrombogenic material (e.g., collagen, laminins, nidogens) into the bloodstream, which ultimately activates the coagulation process [21]. To prevent blood clot formation, ECs are able to balance vascular tone by producing several factors that improve dilatation of muscular arteries. Among these, the most important are NO and prostaglandin I2 (PGI_2_), which combine both antiaggregatory and vasodilator effect [22].

ECs express on their surface a large concentration of molecules involved in the activation of anticoagulant pathways, among which heparan sulphate promotes the anticoagulant effect of antithrombin III (ATIII), while thrombomodulin (TM) stimulates protein C and protein S function [23]. The endothelium can also express plasminogen activators, such as tissue-type plasminogen activator (tPA) and urokinase plasminogen activator (uPA), which enhance the fibrinolytic processes [24,25]. Moreover, ECs can produce adhesion molecules for platelets, such as von Willebrand factor (vWF) and P-selectin, which are exposed on ECs surface upon activation by IL-1β and TNF-α [23]. In turn, platelets produce vascular endothelial growth factor (VEGF), which stimulates the production of tissue factor (TF) from ECs, thus enhancing the activation of coagulation cascade [26].

## 3. Endothelial Function Assessment

Considering its potential reversibility with targeted strategies, several clinical and laboratory methods have been proposed to evaluate and monitor endothelial function, both in humans and in animal models.

### 3.1. Clinical Methods

**Figure 1:**
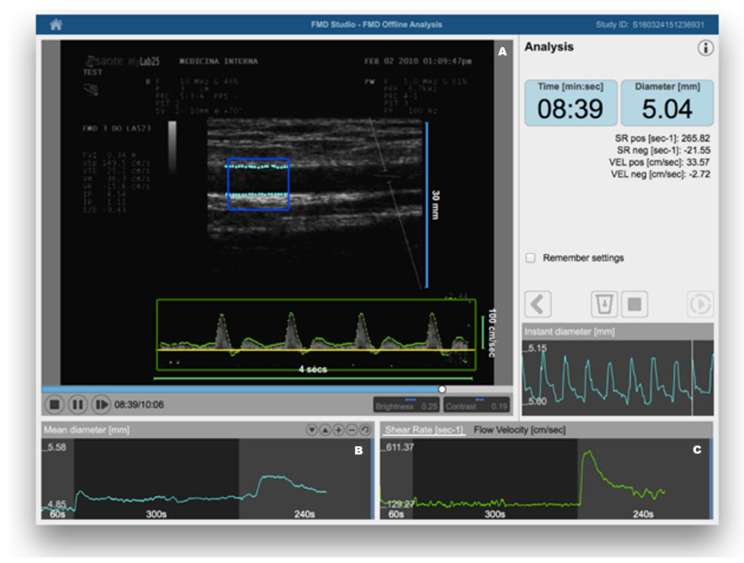
Flow-mediated dilation (FMD) assessment using a Food and Drug Administration (FDA)-cleared software for automatic edge detection (Panel (**A**)), wall tracking (Panel (**B**)), and shear-rate monitoring (Panel (**C**)). Reproduced with permission from Quipu SRL, Pisa, Italy.

FMD was introduced in clinical research about 20 years ago [11]. In brief, it consists of the measurement of changes in brachial artery diameter as a response to shear stress. In order to evoke this response, a pneumatic cuff placed on the forearm is inflated to a suprasystolic pressure for 5 min. When the cuff is deflated, the increased flow enhances the shear stress on the arterial wall, which stimulates the local production of NO, determining vasodilatation [27]. FMD is a measure of the percentage change of the brachial artery diameter after cuff deflation. Much scientific evidence has demonstrated that FMD represents a reliable method for predicting preclinical CV risk [28,29]. Therefore, recognizing endothelial dysfunction could help physicians in early identification of high-risk patients, giving a more comprehensive assessment of CV risk, which may consequently contribute to better evaluation of personalized CV prevention strategies. The recent identification of age- and sex-specific reference values of FMD in healthy subjects has further confirmed the potential clinical utility of its assessment [12]. On the other hand, despite being a non-invasive and inexpensive method, it has been observed that in the same study population there can be large variations of mean FMD values, depending on some technical variables (e.g., occlusion time, cuff position, patient preparation for examination) and the subsequent operator-dependence [30]. When identifying their reference intervals of FMD, Holder et al. highlighted the need for strict adherence to standardized protocols [12]. However, this may not be sufficient. Thus, the use of dedicated software for real-time edge detection, wall tracking, and shear-rate monitoring has proven to significantly increase reproducibility [31] (Figure 1).

Other clinical methods have been proposed for clinical assessment of endothelial function. While venous occlusion plethysmography (VOP) is largely underused because of its invasiveness, laser Doppler flowmetry (LDF) has been used as a non-invasive clinical method for measurement of endothelium-dependent vasodilation in the skin microcirculation [32]. More recently, peripheral artery tonometry (PAT) has become a Food and Drug Administration (FDA)-approved test for an automated assessment of endothelial function [33]. However, although less operator-dependent and highly reproducible, these methods have the disadvantage of being more expensive to use in routine clinical practice and, sometimes, even for research purposes [34].

### 3.2. Laboratory Methods

Taken together, clinical tests allow measurement of microvascular and macrovascular reactivity, which may fully or partially reflect NO bioavailability. However, a healthy endothelium does not only display a vasodilatory phenotype, depending mainly on NO synthesis [32]. As widely discussed below, under normal circumstances, the endothelium also has an anticoagulant phenotype, which is reflected in the constitutive expression of plasminogen activator inhibitor-1 (PAI-1), vWF, and TF, whose soluble forms can be measured in peripheral blood [35]. The endothelium is also responsible for control of inflammation and oxidative stress, with healthy individuals having low levels of soluble endothelium-derived adhesion molecules or chemokines, including ICAM-1, VCAM-1, E-selectin, P-selectin, VE-cadherin, and monocyte chemotactic protein-1 (MCP-1) [35]. More recently, the levels of various components of the glycocalyx (e.g., syndecan-1, endocan, and heparan sulfate) have been proposed as markers of endothelial injury [36]. Moreover, endothelial progenitor cells (EPCs), reflecting vascular repair capacity, are detected in the blood of healthy individuals, with a progressive reduction with aging and various quantitative and functional alterations in response to acute or chronic pathological stimuli [37]. On the other hand, circulating endothelial cells (CECs) and endothelial microparticles (EMPs) are usually low in healthy individuals, since they reflect the presence of endothelial injury [38]. Overall, a plethora of endothelial biomarkers have been widely used for the identification and characterization of specific endothelial cell types and to test endothelial function both in humans and in animal models.

## 4. Evidence of Endothelial Dysfunction in COVID-19

From the early stages of the pandemic, it has emerged that endothelial dysfunction could represent the unifying mechanism of COVID-19. Varga et al. were among the first to perform histopathological examinations from autoptic specimens, confirming the presence of endotheliitis in many organs and tissues, with electron microscopy also revealing the presence of SARS-CoV-2 within ECs [6]. The involvement of ECs in the kidneys, lung, heart, skin, and even reproductive system was subsequently highlighted in multiple studies [39,40], suggesting that endothelial damage could represent an important pathogenetic mechanism of respiratory and multiorgan dysfunction [41,42], with a variety of manifestations ranging from CV complications to adverse perinatal outcomes or even erectile dysfunction [43,44].

Using both clinical and laboratory methods for endothelial function assessment, mounting evidence has confirmed the presence of endothelial dysfunction related to SARS-CoV-2 infection. Summarizing the current evidence, a recent meta-analysis showed that several biomarkers of endothelial dysfunction, including vWF, tPA, PAI-1, and soluble thrombomodulin, are significantly associated with increased composite poor outcomes in patients with COVID-19 [45]. Similarly, in addition to these circulatory markers of endothelial function, another meta-analysis showed that high circulating levels of VCAM-1 and E-selectin may be associated with increased COVID-19 severity [46]. Mancuso et al. were among the first to suggest the monitoring of CECs and EPCs as candidate biomarkers of endothelial damage in COVID-19 patients [47]. More recently, increased production of EPCs was also demonstrated during convalescence [48].

Applying the ESC recommendations [4], a number of studies also used clinical methods to test and monitor endothelial function in COVID-19 patients, particularly after the acute phase [7,14,49]. As stated above, most studies employed FMD, given its cost-effectiveness and non-invasiveness, but only a small percentage resorted to dedicated edge-detection software. In the largest study on this topic [7], significantly lower FMD was documented in convalescent COVID-19 patients as compared to controls, confirmed when stratifying the study population according to age and major clinical variables. However, no significant difference was observed between cases and controls in the subgroup analysis on females, in line with the evidence of a disproportionately worse prognosis for male gender [50]. Similar findings were documented among six COVID-19 patients without CV risk when using PAT for endothelial function assessment [51].

## 5. Pathophysiology of Endothelial Dysfunction in COVID-19

In COVID-19 patients, the dysregulation of many of the homeostatic pathways has emerged as a mediator of severe disease [52]. Therefore, COVID-19 was ultimately described as an endothelial disease [17]. Several hypotheses have been proposed to explain endotheliopathy in this clinical setting, involving both direct and indirect viral actions.

### 5.1. Direct Viral Action

To enter the cells, it has been proven that SARS-CoV-2 is able to bind angiotensin-converting enzyme 2 (ACE2), normally expressed on human cells, helped by the transmembrane protease serine 2 (TMPRSS2) [7]. Thus, it is reasonable to affirm that human cells expressing ACE2 and TMPRSS2 on their surface represent SARS-CoV-2 target cells [7]. In this regard, there is evidence that ECs show a large concentration of ACE2 on their surface [53], so they may ideally represent the natural target for the infection. Accordingly, several reports documented the presence of SARS-CoV-2 within ECs in various organs and tissues [6,39], thus potentially activating endothelial apoptotic pathways. This is consistent with the high plasma levels of Tie-2 receptor and syndecan-1 in critical COVID-19 patients, reflecting the rupture of the endothelial glycocalyx covering the luminal surface of ECs [54]. However, the capacity of the virus to directly infect the endothelium has recently been put into question [55,56], in line with the evidence that ECs derived from human pluripotent stem cells have been shown to be resistant to SARS-CoV-2 infection [57]. Despite these contrasting findings, the hypothesis of a direct viral infection of ECs may—at least in part—be supported by the evidence that females may be more resistant to the deleterious effects of SARS-CoV-2, including endothelial dysfunction [7].

It is well known that the ACE2 gene is an “escape gene” localized in the Xp22.2 region of the X chromosome [58], thus being resistant to chromatin inactivation [59]. Consequently, it can be argued that females have a “double dose” of ACE2 [60], with estrogens also having the capacity to upregulate ACE2 expression [61]. This may counteract the downregulation of ACE2, due to the endocytosis of the enzyme along with the viral particles [62], and due to the inflammatory upregulation of a disintegrin and metalloproteinase 17 (ADAM17) deputized to the proteolytic cleavage of ACE2 [7]. It is important to highlight that ACE2 is not only the key to entry for SARS-CoV-2 to human cells. One of its main functions is converting angiotensin II to angiotensin_1-7_, with this degradation peptide having several counter-regulatory effects on angiotensin II [7]. Angiotensin II is able to decrease endothelial NO phosphorylation, thus leading to reduced NO synthesis [63]. This effect is mediated by the angiotensin II type 1 (AT1) receptor coupled to the Gα12/13 family of G proteins, with the involvement of a RhoA/Rho kinase pathway and the activation of the p38 mitogen-activated protein kinase (MAPK) [7]. Both G protein and non-G protein signaling cascades following AT1 binding may determine increased oxidative stress, with the activation of the nicotinamide adenine dinucleotide phosphate (NADPH) oxidase and the production of reactive oxygen species (ROS), including superoxide anion (O_2_^−^), hydrogen peroxide (H_2_O_2_), and peroxynitrite [64]. The excess of ROS may in turn initialize a number of additional molecular pathways, which finally stimulate the synthesis of inflammatory cytokines (IL-1β, IL-6, and TNF-α), induce EC apoptosis, and reduce NO bioavailability [64]. Moreover, angiotensin II is able to directly stimulate inflammation by activating nuclear factor-κB (NF-κB), thus enhancing the transcription of inflammatory cytokines and adhesion molecules as well as collagen deposition and the overexpression of endothelin-1 and PAI-1 [65]. This may ultimately account for the increased thrombotic risk.

### 5.2. Indirect Viral Action

**Figure 2:**
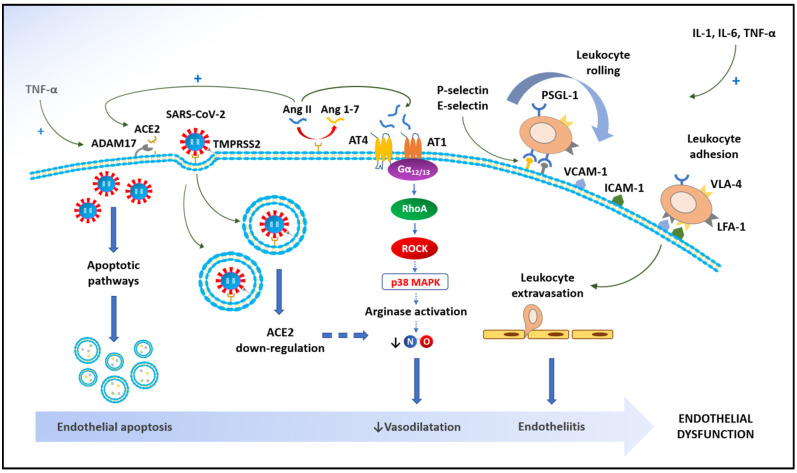
Physiopathology of endothelial dysfunction in coronavirus disease 2019 (COVID-19). SARS-CoV-2: severe acute respiratory syndrome coronavirus 2; TNF-α: tumor necrosis factor alpha; ADAM17: a disintegrin and metalloprotease 17; ACE2: angiotensin-converting enzyme 2; Ang II: angiotensin II; Ang 1-7: angiotensin_1-7_; AT1: angiotensin receptor type 1; AT4: angiotensin receptor type 4; Gα_12/13_: guanine nucleotide-binding protein alpha 12/13; RhoA: Ras homolog family member A; ROCK: Rho-associated protein kinase; p38 MAPK: p38 mitogen-activated protein kinase; NO: nitric oxide; IL-1: interleukin-1; IL-6: interleukin-6; TNF-α: tumor necrosis factor-α; VCAM-1: vascular cell adhesion molecule-1; ICAM-1: intercellular adhesion molecule-1; PSGL-1: P-selectin glycoprotein ligand-1; VLA-4: very late antigen-4 integrin; LFA-1: lymphocyte function-associated antigen-1 integrin.

Beyond a direct effect on ECs, it is reasonable to assume that endothelial dysfunction is also the consequence of systemic inflammation. The pathophysiological mechanisms underlying the massive inflammatory systemic response to SARS-CoV-2 infection have been well-studied [66]. In brief, viral infection of host cells may lead to the release of proinflammatory cytokines [67] through the recognition mechanisms of the innate immune response (i.e., pattern recognition receptors (PRRs), toll-like receptors (TLRs), and NOD-like receptors (NLRs)), thus allowing the identification of intracellular viral RNA [5]. The recognition of viral RNA by these receptors activates a number of intracellular signaling pathways, including NF-κB, which ultimately results in the transcription of proinflammatory cytokines, such as IL-1β, IL-6, and TNF-α [5]. In this process, IL-1β seems to play a pivotal role, since it upregulates its own gene expression in ECs, stimulating the local production of chemokines, which in turn regulates the penetration of inflammatory cells into tissues [68]. IL-1β also induces the secretion of another proinflammatory cytokine, namely IL-6, which leads to the amplification of the systemic inflammatory response through cytokine overproduction [69]. IL-6 provides a large contribution to endothelial dysfunction in COVID-19 patients. In fact, through JAK-STAT activation, IL-6 enhances the expression of adhesion molecules on EC surface (i.e., VCAM-1, ICAM-1, and E-selectin), thus promoting the recruitment of leucocytes into the vascular wall while reducing NO bioavailability and increasing oxidative stress via activation of NADPH oxidase [21,23]. Contrary to IL-6, which mainly acts through JAK-STAT activation, TNF-α transcriptional activity substantially depends on NF-κB [70]. Among a plethora of actions on ECs, including the overexpression of adhesion molecules, TNF-α downregulates VE-cadherin expression and stimulates phosphorylation of tyrosine in VE-cadherin [70]. This ultimately results in the disruption of its contact with beta-catenin and subsequently enhances endothelial permeability [71]. Moreover, inflammatory cytokines lead to a simultaneous increase in the expression of vWF and TF on ECs, which will promote blood clotting through the increase in platelet aggregation and the initiation of the coagulation cascade, respectively [72]. Overall, the presence of these indirect mechanisms of endothelial dysfunction, mediated by the systemic inflammatory response, may justify the systemic involvement of the endothelium in COVID-19 patients [4] (Figure 2).

### 5.3. Potential Inflammatory Mechanisms from Other Respiratory Diseases

Patients with chronic obstructive pulmonary disease (COPD) have an increased risk of severe pneumonia and poor outcomes when they develop COVID-19 [73]. Cigarette smoke and COPD have been associated with higher ACE2 expression in the lungs and it has been hypothesized that this may increase SARS-CoV-2 infection susceptibility [74]. On the other hand, COVID-19 could represent the ultimate cause of acute exacerbation in COPD patients [74]. Thus, the strict clinical interrelationship between these two respiratory conditions led to the hypothesis that the mechanisms of pulmonary endothelial damage in COPD may also be somehow involved in SARS-CoV-2 infection [74].

In COPD, the recruited leucocytes (cytotoxic CD8+ T cells, neutrophils, monocytes, and B cells) sustain chronic local inflammation leading to hypoxia, vasoconstriction, and injury of extracellular matrix and endothelial lining [75]. The first microscopic alteration in endothelium involves the basement membrane, which becomes thicker than in healthy individuals [75], later becoming fragmented and more vascularized [76,77]. It has also been observed that in COPD patients, apoptotic ECs appear in the vascular wall, presenting fragmented nucleoli and leading to enhanced matrix metalloproteinases (MMPs) activity, so that the alveolar wall loses its elasticity and collapses [78,79]. It has been demonstrated that neutrophils are able to generate novel cellular processes such as the neutrophil extracellular trap (NET) [80], thus attaching to the apical domain of ECs and then migrating by their pseudopods over one (transendothelial migration, TEM) or between two ECs (paracellular transmigration, PCM) [81]. Recently, a reverse TEM (rTEM) has also been observed: some neutrophils migrate from abluminal-to-luminal direction through ECs, thus mediating the systemic dissemination of inflammation, while other neutrophils reverse back in the interstitial space [82]. Here, lung-resident macrophages increase in number and together with neutrophils induce degradation of extracellular matrix (ECM), oxidative stress, apoptosis, expression of surface intracellular markers, and dysregulated activation of proinflammatory mediators and proteases [83]. Overall, these molecular changes induce a decreased oxygen level in the lung tissue and the subsequent activation of hypoxia-induced factors (HIF) as well as the transcription of platelet-derived growth factor-β (PDGF-β). HIF-1 induces inflammation, while HIF-2 induces expression of endothelin-1 and arginase (potent vasoconstrictors) in ECs, while downregulating apelin expression, a molecule involved in vasodilatation [84]. Moreover, HIF-1α regulates VEGF, inducing its overexpression together with that of its receptors (VEGFRs). VEGF and VEGFRs are involved in angiogenesis in COPD, correlating with its severity because the reduction in the number of certain VEGF isoforms seems to correlate with apoptosis responsible for alveolar septa of emphysematous lungs [85].

Overall, chronic impairment of the innate and acquired immune responses at least results in delayed viral clearance in COPD patients, thus potentially favoring SARS-CoV-2 infection [74]. However, whether and to what extent the aforementioned mechanisms that have been well-studied in COPD may also be involved in the pathogenesis of COVID-19 alterations is yet to be determined. It is reasonable to assume that, since they reflect the parenchymal modifications of a chronic condition with a different etiology, they may not be involved in the acute phase but rather contribute—at least in a minimal way—to the long-term manifestations that have been documented in COVID-19 survivors. Recently, particular attention has been given to the capacity of hypoxic stress and subsequent HIF upregulation to activate COPD-like mechanisms in COVID-19 [86]. However, further laboratory and translational studies are needed to better address this issue.

## 6. Targeting Endothelial Dysfunction in COVID-19

Considering its systemic nature and reversibility in early stages, endothelial dysfunction has been proposed as a therapeutic target in different clinical settings [87]. Although most therapeutic strategies for COVID-19 have focused so far on the suppression of viral replication, it can be argued that targeting endothelial dysfunction may also represent an additional and attractive strategy in this clinical setting [88]. Two main classes of drugs, namely renin-angiotensin system (RAS) inhibitors and statins, have previously shown a positive impact on endothelial function in terms of vascular tone and coagulation control [89]. However, other pharmacological and non-pharmacological strategies have shown promise in countering endothelial dysfunction [90].

### 6.1. RAS Inhibitors

The use of RAS inhibitors, including ACE inhibitors and angiotensin receptor blockers (ARBs) is controversial in COVID-19 patients. In other clinical settings, they have already shown the capacity to improve endothelial function [91], thus also reducing the thrombotic risk, given their capacity to reduce TF expression [92]. However, RAS inhibitors are able to upregulate ACE2 expression [93], thereby theoretically increasing susceptibility to the virus. On the other hand, as widely discussed above, ACE2 is not only the key to entry for SARS-CoV-2 into human cells. One of its main functions is converting angiotensin II to angiotensin_1-7_, which has several counter-regulatory effects on angiotensin II (e.g., NO release, antifibrotic, anti-inflammatory, anticoagulant).

In a large observational study in England [94], the use of ACE inhibitors and ARBs did not increase the risk of intensive care unit (ICU) admittance, while significantly reducing the risk of COVID-19 among over 8 million participants. Accordingly, no impact on mortality risk was found when specifically considering hospitalized patients [95,96]. A meta-analysis of observational studies showed instead that patients with COVID-19 using RAS inhibitors had a significantly lower risk of death than those who did not [97]. The first randomized controlled trial (RCT) on this topic, namely BRACE-CORONA, suggested that the discontinuation of RAS inhibitors in hospitalized COVID-19 patients did not have any additional beneficial effect [98]. In contrast with their previous meta-analytical data on observational evidence [97], the same research group recently published a meta-analysis of RCT, revealing no difference in mortality risk between COVID-19 patients with or without RAS inhibitors [99].

Overall, current evidence appears to suggest that the use of RAS inhibitors should not be discontinued in COVID-19 patients, as they may not have deleterious effects on the course of COVID-19. On the other hand, since ACE2 is both the virus gateway and an important RAS component, there is no evidence to recommend their use to treat endothelial dysfunction in COVID-19.

### 6.2. Statins

Statins may represent another promising pharmacological strategy to target endothelial dysfunction in this clinical setting [100]. Similar to RAS inhibitors, the pathophysiological mechanisms through which statins protect the endothelium are multiple, including the prevention of endothelial NO synthase uncoupling, the inhibition of NF-κB and other inflammatory pathways, and the reduction in TF expression with subsequent anticoagulant effect [89]. In the framework of comprehensive reduction of CV risk, another mechanism of action is the reduction of low-density lipoprotein (LDL) cholesterol, thus contrasting the LDL-induced endothelial dysfunction and oxidative stress [101]. These potentially beneficial effects are confirmed by the meta-analytical evidence that chronic statin use is associated with lower mortality in COVID-19 patients [102,103]. Again, the evidence from RCTs appears to be completely different, with statins being safe in COVID-19 but unable to change the outcome [104]. On the other hand, the INSPIRATION-S investigators also documented that statin treatment may be beneficial in the early phases of the disease, within 7 days from ICU admission, probably before the inflammatory response leads to irreversible damage [105].

### 6.3. Antioxidants and Other Pharmacological Strategies

A number of other pharmacological strategies have been investigated to treat COVID-19 and to prevent the most severe evolution of the disease. Most of them, including corticosteroids, heparin, serine protease inhibitors, and biological agents targeting inflammatory cytokines (e.g., IL-1, IL-6, TNF-α) or their receptors, have the ability to act at least in part by counteracting endothelial dysfunction and the imbalanced prothrombotic properties of the endothelium through direct or indirect mechanisms [106,107].

Since SARS-CoV-2 infection triggers oxidative stress [108], antioxidant therapies have also been proposed for COVID-19 patients. Given the strict interrelationship between oxidative stress and endothelial dysfunction, strategies targeting oxidative stress may also be useful in improving endothelial function [109]. Dimethylfumarate is approved for the treatment of multiple sclerosis and psoriasis and is also a strong activator of the nuclear factor erythroid 2-related factor 2 (Nrf2), a well-known antioxidant transcription factor that restores cellular redox homeostasis [110]. Its use has been proposed for COVID-19, given its ability to inhibit TMPRSS2, thus limiting the entry of the virus [111]. Sulforaphane is another potent activator of Nrf2, which is currently being tested in several clinical trials on COPD and has also shown its potential utility in COVID-19 [112]. Glutathione and N-acetyl cysteine (NAC), a precursor of glutathione, are potent antioxidants involved in the removal of H_2_O_2_ and other ROS [113]. Moreover, NAC has anticoagulant properties and provides protection against the deleterious effects of angiotensin II, since it inhibits ACE2 receptors [114]. For these reasons, these compounds have also been tested in COVID-19 with preliminary encouraging results [115,116]. Other antioxidant therapies, including vitamins (e.g., C, D, and E) and zinc, which have already been shown to improve endothelial function in other clinical settings [117], may lead to an improvement of respiratory symptoms during SARS-CoV-2 infection [118]. Being a precursor of NO, L-arginine is another compound that potentially plays a role in counteracting oxidative stress and endothelial dysfunction in COVID-19 [119]. L-arginine has already proven its safety and efficacy in patients with severe COVID-19, significantly reducing hospitalization length and the need for respiratory support [120]. However, it has not been studied whether it could be effective in improving endothelial function and CV risk, particularly in the convalescent phase. Overall, randomized pharmacological studies should elucidate the real utility of these compounds in COVID-19.

### 6.4. Rehabilitation and Exercise-Based Approaches

Another therapeutic approach, namely rehabilitation, which has already shown utility in COVID-19, may target endothelial dysfunction following the acute phase, thus potentially reducing CV risk during convalescence. From the early stages of the pandemic, it was hypothesized that COVID-19 could leave behind a plethora of clinical and functional sequelae, not only in the lungs but at a systemic level [3]. Therefore, the possible usefulness of rehabilitation strategies to reduce the psychological, CV, and respiratory consequences of the disease has been hypothesized from the beginning [121]. The evidence from recent RCTs [122,123] and observational studies [124,125] suggest that exercise-based rehabilitation in convalescent COVID-19 patients may be effective in improving symptoms, quality of life, pulmonary function, and even computed tomography (CT) abnormalities.

In 1986, Sinoway et al. were the first to demonstrate that exercise may improve endothelial function in tennis players [126]. This was also shown in a number of studies focusing on rehabilitation and other exercise-based interventions both in healthy subjects and in different clinical settings (e.g., COPD, heart failure) [127,128,129,130], later confirmed by recent meta-analytical evidence [131]. A number of mechanisms have been called into question to explain the beneficial effects of exercise on endothelial function, including upregulation of superoxide dismutase, increased endothelial NO synthase phosphorylation and reduced uncoupling, downregulation of NADPH oxidase, and EPC mobilization [132]. In COVID-19, we were the first to suggest the potential usefulness of exercise-based rehabilitation in reducing endothelial dysfunction, with the improvement in FMD being positively correlated with the improvement in pulmonary function. However, this was only preliminary evidence, given the observational design, the lack of a control group, and the absence of concomitant laboratory testing of endothelial function [88]. Large well-designed observational studies with a controlled design focusing on both clinical and laboratory biomarkers of endothelial function are warranted to clarify the possibility of restoring endothelial integrity through exercise and different rehabilitation approaches.

## 7. Conclusions

This review summarized the large amount of scientific evidence, which seems to suggest that endothelial dysfunction represents the common denominator of most clinical manifestations of COVID-19, both in the acute phase and during convalescence. This may be supported by the strong interrelationship between inflammation, oxidative stress, and endothelial function. Although current therapeutic strategies in the acute phase are mainly focused on blocking viral replication and limiting inflammation, it is likely that novel approaches aimed at counteracting endothelial dysfunction could represent a valid option, particularly in a convalescent phase. Further evidence is urgently needed to elucidate the role of statins, RAS inhibitors, antioxidants, rehabilitation, and exercise-based interventions in this clinical setting.

## Data Availability

No datasets were generated or analyzed during the current study.

## References

[1] Guan W.J., Ni Z.Y., Hu Y., Liang W.H., Ou C.Q., He J.X., Liu L., Shan H., Lei C.L., Hui D.S.C. (2020). Clinical Characteristics of Coronavirus Disease 2019 in China. N. Engl. J. Med..

[2] Jin Y., Yang H., Ji W., Wu W., Chen S., Zhang W., Duan G. (2020). Virology, Epidemiology, Pathogenesis, and Control of COVID-19. Viruses.

[3] Ambrosino P., Papa A., Maniscalco M., Di Minno M.N.D. (2021). COVID-19 and functional disability: Current insights and rehabilitation strategies. Postgrad. Med. J..

[4] Evans P.C., Rainger G.E., Mason J.C., Guzik T.J., Osto E., Stamataki Z., Neil D., Hoefer I.E., Fragiadaki M., Waltenberger J. (2020). Endothelial dysfunction in COVID-19: A position paper of the ESC Working Group for Atherosclerosis and Vascular Biology, and the ESC Council of Basic Cardiovascular Science. Cardiovasc. Res..

[5] Fodor A., Tiperciuc B., Login C., Orasan O.H., Lazar A.L., Buchman C., Hanghicel P., Sitar-Taut A., Suharoschi R., Vulturar R. (2021). Endothelial Dysfunction, Inflammation, and Oxidative Stress in COVID-19-Mechanisms and Therapeutic Targets. Oxid. Med. Cell. Longev..

[6] Varga Z., Flammer A.J., Steiger P., Haberecker M., Andermatt R., Zinkernagel A.S., Mehra M.R., Schuepbach R.A., Ruschitzka F., Moch H. (2020). Endothelial cell infection and endotheliitis in COVID-19. Lancet.

[7] Ambrosino P., Calcaterra I., Molino A., Moretta P., Lupoli R., Spedicato G.A., Papa A., Motta A., Maniscalco M., Di Minno M.N.D. (2021). Persistent Endothelial Dysfunction in Post-Acute COVID-19 Syndrome: A Case-Control Study. Biomedicines.

[8] Cuker A., Tseng E.K., Nieuwlaat R., Angchaisuksiri P., Blair C., Dane K., Davila J., DeSancho M.T., Diuguid D., Griffin D.O. (2021). American Society of Hematology 2021 guidelines on the use of anticoagulation for thromboprophylaxis in patients with COVID-19. Blood Adv..

[9] Di Minno A., Ambrosino P., Calcaterra I., Di Minno M.N.D. (2020). COVID-19 and Venous Thromboembolism: A Meta-analysis of Literature Studies. Semin. Thromb. Hemost..

[10] Madjid M., Safavi-Naeini P., Solomon S.D., Vardeny O. (2020). Potential Effects of Coronaviruses on the Cardiovascular System: A Review. JAMA Cardiol..

[11] Corretti M.C., Anderson T.J., Benjamin E.J., Celermajer D., Charbonneau F., Creager M.A., Deanfield J., Drexler H., Gerhard-Herman M., Herrington D. (2002). Guidelines for the ultrasound assessment of endothelial-dependent flow-mediated vasodilation of the brachial artery: A report of the International Brachial Artery Reactivity Task Force. J. Am. Coll. Cardiol..

[12] Holder S.M., Bruno R.M., Shkredova D.A., Dawson E.A., Jones H., Hopkins N.D., Hopman M.T.E., Bailey T.G., Coombes J.S., Askew C.D. (2021). Reference Intervals for Brachial Artery Flow-Mediated Dilation and the Relation With Cardiovascular Risk Factors. Hypertension.

[13] Inaba Y., Chen J.A., Bergmann S.R. (2010). Prediction of future cardiovascular outcomes by flow-mediated vasodilatation of brachial artery: A meta-analysis. Int. J. Cardiovasc. Imaging.

[14] Ergul E., Yilmaz A.S., Ogutveren M.M., Emlek N., Kostakoglu U., Cetin M. (2022). COVID 19 disease independently predicted endothelial dysfunction measured by flow-mediated dilatation. Int. J. Cardiovasc. Imaging.

[15] Tu T.M., Seet C.Y.H., Koh J.S., Tham C.H., Chiew H.J., De Leon J.A., Chua C.Y.K., Hui A.C., Tan S.S.Y., Vasoo S.S. (2021). Acute Ischemic Stroke During the Convalescent Phase of Asymptomatic COVID-2019 Infection in Men. JAMA Netw. Open.

[16] d’Alessandro E., Becker C., Bergmeier W., Bode C., Bourne J.H., Brown H., Buller H.R., Ten Cate-Hoek A.J., Ten Cate V., van Cauteren Y.J.M. (2020). Thrombo-Inflammation in Cardiovascular Disease: An Expert Consensus Document from the Third Maastricht Consensus Conference on Thrombosis. Thromb. Haemost..

[17] Libby P., Luscher T. (2020). COVID-19 is, in the end, an endothelial disease. Eur. Heart J..

[18] Giannotta M., Trani M., Dejana E. (2013). VE-cadherin and endothelial adherens junctions: Active guardians of vascular integrity. Dev. Cell.

[19] Noels H., Weber C., Koenen R.R. (2019). Chemokines as Therapeutic Targets in Cardiovascular Disease. Arterioscler. Thromb. Vasc. Biol..

[20] Mestas J., Ley K. (2008). Monocyte-endothelial cell interactions in the development of atherosclerosis. Trends Cardiovasc. Med..

[21] Sturtzel C. (2017). Endothelial Cells. Adv. Exp. Med. Biol..

[22] Furchgott R.F. (1999). Endothelium-Derived Relaxing Factor: Discovery, Early Studies, and Identifcation as Nitric Oxide (Nobel Lecture). Angew. Chem. Int. Ed. Engl..

[23] Pober J.S., Sessa W.C. (2007). Evolving functions of endothelial cells in inflammation. Nat. Rev. Immunol..

[24] Sawdey M.S., Loskutoff D.J. (1991). Regulation of murine type 1 plasminogen activator inhibitor gene expression in vivo. Tissue specificity and induction by lipopolysaccharide, tumor necrosis factor-alpha, and transforming growth factor-beta. J. Clin. Investig..

[25] Levin E.G., Loskutoff D.J. (1982). Cultured bovine endothelial cells produce both urokinase and tissue-type plasminogen activators. J. Cell Biol..

[26] Nachman R.L., Rafii S. (2008). Platelets, petechiae, and preservation of the vascular wall. N. Engl. J. Med..

[27] Thijssen D.H., Black M.A., Pyke K.E., Padilla J., Atkinson G., Harris R.A., Parker B., Widlansky M.E., Tschakovsky M.E., Green D.J. (2011). Assessment of flow-mediated dilation in humans: A methodological and physiological guideline. Am. J. Physiol. Heart Circ. Physiol..

[28] Celermajer D.S., Sorensen K.E., Bull C., Robinson J., Deanfield J.E. (1994). Endothelium-dependent dilation in the systemic arteries of asymptomatic subjects relates to coronary risk factors and their interaction. J. Am. Coll. Cardiol..

[29] Anderson T.J. (2007). Prognostic significance of brachial flow-mediated vasodilation. Circulation.

[30] Bots M.L., Westerink J., Rabelink T.J., de Koning E.J. (2005). Assessment of flow-mediated vasodilatation (FMD) of the brachial artery: Effects of technical aspects of the FMD measurement on the FMD response. Eur. Heart J..

[31] Greyling A., van Mil A.C., Zock P.L., Green D.J., Ghiadoni L., Thijssen D.H., Dilation T.I.W.G.o.F.M. (2016). Adherence to guidelines strongly improves reproducibility of brachial artery flow-mediated dilation. Atherosclerosis.

[32] Klonizakis M., Manning G., Donnelly R. (2011). Assessment of lower limb microcirculation: Exploring the reproducibility and clinical application of laser Doppler techniques. Skin Pharmacol. Physiol..

[33] Rubinshtein R., Kuvin J.T., Soffler M., Lennon R.J., Lavi S., Nelson R.E., Pumper G.M., Lerman L.O., Lerman A. (2010). Assessment of endothelial function by non-invasive peripheral arterial tonometry predicts late cardiovascular adverse events. Eur. Heart J..

[34] Flammer A.J., Anderson T., Celermajer D.S., Creager M.A., Deanfield J., Ganz P., Hamburg N.M., Luscher T.F., Shechter M., Taddei S. (2012). The assessment of endothelial function: From research into clinical practice. Circulation.

[35] Goncharov N.V., Nadeev A.D., Jenkins R.O., Avdonin P.V. (2017). Markers and Biomarkers of Endothelium: When Something Is Rotten in the State. Oxid. Med. Cell. Longev..

[36] Celik T., Balta S., Karaman M., Ahmet Ay S., Demirkol S., Ozturk C., Dinc M., Unal H.U., Yilmaz M.I., Kilic S. (2015). Endocan, a novel marker of endothelial dysfunction in patients with essential hypertension: Comparative effects of amlodipine and valsartan. Blood Press..

[37] Savoia C., Grassi G. (2012). Exercise activity and endothelial function: The uprising role of endothelial progenitor cells in vascular protection. J. Hypertens..

[38] Sabatier F., Camoin-Jau L., Anfosso F., Sampol J., Dignat-George F. (2009). Circulating endothelial cells, microparticles and progenitors: Key players towards the definition of vascular competence. J. Cell. Mol. Med..

[39] Ackermann M., Verleden S.E., Kuehnel M., Haverich A., Welte T., Laenger F., Vanstapel A., Werlein C., Stark H., Tzankov A. (2020). Pulmonary Vascular Endothelialitis, Thrombosis, and Angiogenesis in COVID-19. N. Engl. J. Med..

[40] Kolivras A., Dehavay F., Delplace D., Feoli F., Meiers I., Milone L., Olemans C., Sass U., Theunis A., Thompson C.T. (2020). Coronavirus (COVID-19) infection-induced chilblains: A case report with histopathologic findings. JAAD Case Rep..

[41] Dou Q., Wei X., Zhou K., Yang S., Jia P. (2020). Cardiovascular Manifestations and Mechanisms in Patients with COVID-19. Trends Endocrinol. Metab..

[42] Kazemi S., Pourgholaminejad A., Saberi A. (2021). Stroke Associated with SARS-CoV-2 Infection and its Pathogenesis: A Systematic Review. Basic Clin. Neurosci..

[43] Pathirathna M.L., Samarasekara B.P.P., Dasanayake T.S., Saravanakumar P., Weerasekara I. (2022). Adverse Perinatal Outcomes in COVID-19 Infected Pregnant Women: A Systematic Review and Meta-Analysis. Healthcare.

[44] Delli Muti N., Finocchi F., Tossetta G., Salvio G., Cutini M., Marzioni D., Balercia G. (2022). Could SARS-CoV-2 infection affect male fertility and sexuality?. APMIS.

[45] Andrianto, Al-Farabi M.J., Nugraha R.A., Marsudi B.A., Azmi Y. (2021). Biomarkers of endothelial dysfunction and outcomes in coronavirus disease 2019 (COVID-19) patients: A systematic review and meta-analysis. Microvasc. Res..

[46] Lampsas S., Tsaplaris P., Pantelidis P., Oikonomou E., Marinos G., Charalambous G., Souvaliotis N., Mystakidi V.C., Goliopoulou A., Katsianos E. (2021). The Role of Endothelial Related Circulating Biomarkers in COVID-19. A Systematic Review and Meta-analysis. Curr. Med. Chem..

[47] Mancuso P., Gidaro A., Gregato G., Raveane A., Cremonesi P., Quarna J., Caccia S., Gusso L., Rusconi S., Giacomelli A. (2020). Circulating endothelial progenitors are increased in COVID-19 patients and correlate with SARS-CoV-2 RNA in severe cases. J. Thromb. Haemost..

[48] Poyatos P., Luque N., Eizaguirre S., Sabater G., Sebastian L., Albesa I.F., Peracaula M., Boixade M., Orriols R., Tura-Ceide O. (2022). Post-COVID-19 patients show an increased endothelial progenitor cell production. Transl. Res..

[49] Paneroni M., Pasini E., Vitacca M., Scalvini S., Comini L., Pedrinolla A., Venturelli M. (2021). Altered Vascular Endothelium-Dependent Responsiveness in Frail Elderly Patients Recovering from COVID-19 Pneumonia: Preliminary Evidence. J. Clin. Med..

[50] Grasselli G., Zangrillo A., Zanella A., Antonelli M., Cabrini L., Castelli A., Cereda D., Coluccello A., Foti G., Fumagalli R. (2020). Baseline Characteristics and Outcomes of 1591 Patients Infected With SARS-CoV-2 Admitted to ICUs of the Lombardy Region, Italy. JAMA.

[51] Cimino G., Vizzardi E., Calvi E., Pancaldi E., Pascariello G., Bernardi N., Cersosimo A., Amore L., Inciardi R.M., Raddino R. (2022). Endothelial dysfunction in COVID-19 patients assessed with Endo-PAT2000. Monaldi Arch. Chest Dis..

[52] Siddiqi H.K., Libby P., Ridker P.M. (2021). COVID-19—A vascular disease. Trends Cardiovasc. Med..

[53] Chen L., Li X., Chen M., Feng Y., Xiong C. (2020). The ACE2 expression in human heart indicates new potential mechanism of heart injury among patients infected with SARS-CoV-2. Cardiovasc. Res..

[54] Stahl K., Gronski P.A., Kiyan Y., Seeliger B., Bertram A., Pape T., Welte T., Hoeper M.M., Haller H., David S. (2020). Injury to the Endothelial Glycocalyx in Critically Ill Patients with COVID-19. Am. J. Respir. Crit. Care Med..

[55] Wichmann D., Sperhake J.P., Lutgehetmann M., Steurer S., Edler C., Heinemann A., Heinrich F., Mushumba H., Kniep I., Schroder A.S. (2020). Autopsy Findings and Venous Thromboembolism in Patients With COVID-19: A Prospective Cohort Study. Ann. Intern. Med..

[56] McCracken I.R., Saginc G., He L., Huseynov A., Daniels A., Fletcher S., Peghaire C., Kalna V., Andaloussi-Mae M., Muhl L. (2021). Lack of Evidence of Angiotensin-Converting Enzyme 2 Expression and Replicative Infection by SARS-CoV-2 in Human Endothelial Cells. Circulation.

[57] Yang L., Han Y., Nilsson-Payant B.E., Gupta V., Wang P., Duan X., Tang X., Zhu J., Zhao Z., Jaffre F. (2020). A Human Pluripotent Stem Cell-based Platform to Study SARS-CoV-2 Tropism and Model Virus Infection in Human Cells and Organoids. Cell Stem Cell.

[58] Tukiainen T., Villani A.C., Yen A., Rivas M.A., Marshall J.L., Satija R., Aguirre M., Gauthier L., Fleharty M., Kirby A. (2017). Landscape of X chromosome inactivation across human tissues. Nature.

[59] Berletch J.B., Yang F., Xu J., Carrel L., Disteche C.M. (2011). Genes that escape from X inactivation. Hum. Genet..

[60] Viveiros A., Rasmuson J., Vu J., Mulvagh S.L., Yip C.Y.Y., Norris C.M., Oudit G.Y. (2021). Sex differences in COVID-19: Candidate pathways, genetics of ACE2, and sex hormones. Am. J. Physiol. Heart Circ. Physiol..

[61] Bukowska A., Spiller L., Wolke C., Lendeckel U., Weinert S., Hoffmann J., Bornfleth P., Kutschka I., Gardemann A., Isermann B. (2017). Protective regulation of the ACE2/ACE gene expression by estrogen in human atrial tissue from elderly men. Exp. Biol. Med..

[62] Gagliardi M.C., Tieri P., Ortona E., Ruggieri A. (2020). ACE2 expression and sex disparity in COVID-19. Cell Death Discov..

[63] Satoh M., Fujimoto S., Arakawa S., Yada T., Namikoshi T., Haruna Y., Horike H., Sasaki T., Kashihara N. (2008). Angiotensin II type 1 receptor blocker ameliorates uncoupled endothelial nitric oxide synthase in rats with experimental diabetic nephropathy. Nephrol. Dial. Transplant..

[64] Ding J., Yu M., Jiang J., Luo Y., Zhang Q., Wang S., Yang F., Wang A., Wang L., Zhuang M. (2020). Angiotensin II Decreases Endothelial Nitric Oxide Synthase Phosphorylation via AT1R Nox/ROS/PP2A Pathway. Front. Physiol..

[65] Wolf G., Wenzel U., Burns K.D., Harris R.C., Stahl R.A., Thaiss F. (2002). Angiotensin II activates nuclear transcription factor-κB through AT1 and AT2 receptors. Kidney Int..

[66] Hu B., Huang S., Yin L. (2021). The cytokine storm and COVID-19. J. Med. Virol..

[67] Sansico F., Miroballo M., Bianco D.S., Tamiro F., Colucci M., Santis E., Rossi G., Rosati J., Di Mauro L., Miscio G. (2021). COVID-19 Specific Immune Markers Revealed by Single Cell Phenotypic Profiling. Biomedicines.

[68] Wang J.M., Sica A., Peri G., Walter S., Padura I.M., Libby P., Ceska M., Lindley I., Colotta F., Mantovani A. (1991). Expression of monocyte chemotactic protein and interleukin-8 by cytokine-activated human vascular smooth muscle cells. Arterioscler. Thromb..

[69] Kandere-Grzybowska K., Letourneau R., Kempuraj D., Donelan J., Poplawski S., Boucher W., Athanassiou A., Theoharides T.C. (2003). IL-1 induces vesicular secretion of IL-6 without degranulation from human mast cells. J. Immunol..

[70] Chernyak B.V., Popova E.N., Prikhodko A.S., Grebenchikov O.A., Zinovkina L.A., Zinovkin R.A. (2020). COVID-19 and Oxidative Stress. Biochemistry.

[71] Angelini D.J., Hyun S.W., Grigoryev D.N., Garg P., Gong P., Singh I.S., Passaniti A., Hasday J.D., Goldblum S.E. (2006). TNF-alpha increases tyrosine phosphorylation of vascular endothelial cadherin and opens the paracellular pathway through fyn activation in human lung endothelia. Am. J. Physiol. Lung Cell. Mol. Physiol..

[72] Han H., Yang L., Liu R., Liu F., Wu K.L., Li J., Liu X.H., Zhu C.L. (2020). Prominent changes in blood coagulation of patients with SARS-CoV-2 infection. Clin. Chem. Lab. Med..

[73] Leung J.M., Niikura M., Yang C.W.T., Sin D.D. (2020). COVID-19 and COPD. Eur. Respir. J..

[74] Polverino F., Kheradmand F. (2020). COVID-19, COPD, and AECOPD: Immunological, Epidemiological, and Clinical Aspects. Front. Med..

[75] Szucs B., Szucs C., Petrekanits M., Varga J.T. (2019). Molecular Characteristics and Treatment of Endothelial Dysfunction in Patients with COPD: A Review Article. Int. J. Mol. Sci..

[76] Liesker J.J., Ten Hacken N.H., Zeinstra-Smith M., Rutgers S.R., Postma D.S., Timens W. (2009). Reticular basement membrane in asthma and COPD: Similar thickness, yet different composition. Int. J. Chron. Obstruct. Pulmon. Dis..

[77] Arafah M.A., Raddaoui E., Kassimi F.A., Alhamad E.H., Alboukai A.A., Alshedoukhy A.A., Ouban A. (2018). Endobronchial biopsy in the final diagnosis of chronic obstructive pulmonary disease and asthma: A clinicopathological study. Ann. Saudi Med..

[78] Henson P.M., Vandivier R.W., Douglas I.S. (2006). Cell death, remodeling, and repair in chronic obstructive pulmonary disease?. Proc. Am. Thorac. Soc..

[79] Kraen M., Frantz S., Nihlen U., Engstrom G., Lofdahl C.G., Wollmer P., Dencker M. (2019). Matrix Metalloproteinases in COPD and atherosclerosis with emphasis on the effects of smoking. PLoS ONE.

[80] Bonaventura A., Montecucco F., Dallegri F., Carbone F., Luscher T.F., Camici G.G., Liberale L. (2019). Novel findings in neutrophil biology and their impact on cardiovascular disease. Cardiovasc. Res..

[81] Ayres-Sander C.E., Lauridsen H., Maier C.L., Sava P., Pober J.S., Gonzalez A.L. (2013). Transendothelial migration enables subsequent transmigration of neutrophils through underlying pericytes. PLoS ONE.

[82] Colom B., Bodkin J.V., Beyrau M., Woodfin A., Ody C., Rourke C., Chavakis T., Brohi K., Imhof B.A., Nourshargh S. (2015). Leukotriene B4-Neutrophil Elastase Axis Drives Neutrophil Reverse Transendothelial Cell Migration In Vivo. Immunity.

[83] Kosyreva A., Dzhalilova D., Lokhonina A., Vishnyakova P., Fatkhudinov T. (2021). The Role of Macrophages in the Pathogenesis of SARS-CoV-2-Associated Acute Respiratory Distress Syndrome. Front. Immunol..

[84] Palazon A., Goldrath A.W., Nizet V., Johnson R.S. (2014). HIF transcription factors, inflammation, and immunity. Immunity.

[85] Wang L., Xu Z., Chen B., He W., Hu J., Zhang L., Liu X., Chen F. (2017). The Role of Vascular Endothelial Growth Factor in Small-airway Remodelling in a Rat Model of Chronic Obstructive Pulmonary Disease. Sci. Rep..

[86] Won T., Wood M.K., Hughes D.M., Talor M.V., Ma Z., Schneider J., Skinner J.T., Asady B., Goerlich E., Halushka M.K. (2022). Endothelial thrombomodulin downregulation caused by hypoxia contributes to severe infiltration and coagulopathy in COVID-19 patient lungs. EBioMedicine.

[87] Ambrosino P., Grassi G., Maniscalco M. (2021). Endothelial Dysfunction: From a Pathophysiological Mechanism to a Potential Therapeutic Target. Biomedicines.

[88] Ambrosino P., Molino A., Calcaterra I., Formisano R., Stufano S., Spedicato G.A., Motta A., Papa A., Di Minno M.N.D., Maniscalco M. (2021). Clinical Assessment of Endothelial Function in Convalescent COVID-19 Patients Undergoing Multidisciplinary Pulmonary Rehabilitation. Biomedicines.

[89] Nagele M.P., Haubner B., Tanner F.C., Ruschitzka F., Flammer A.J. (2020). Endothelial dysfunction in COVID-19: Current findings and therapeutic implications. Atherosclerosis.

[90] Daiber A., Steven S., Weber A., Shuvaev V.V., Muzykantov V.R., Laher I., Li H., Lamas S., Munzel T. (2017). Targeting vascular (endothelial) dysfunction. Br. J. Pharmacol..

[91] Shahin Y., Khan J.A., Samuel N., Chetter I. (2011). Angiotensin converting enzyme inhibitors effect on endothelial dysfunction: A meta-analysis of randomised controlled trials. Atherosclerosis.

[92] Napoleone E., Di Santo A., Camera M., Tremoli E., Lorenzet R. (2000). Angiotensin-converting enzyme inhibitors downregulate tissue factor synthesis in monocytes. Circ. Res..

[93] Ishiyama Y., Gallagher P.E., Averill D.B., Tallant E.A., Brosnihan K.B., Ferrario C.M. (2004). Upregulation of angiotensin-converting enzyme 2 after myocardial infarction by blockade of angiotensin II receptors. Hypertension.

[94] Hippisley-Cox J., Young D., Coupland C., Channon K.M., Tan P.S., Harrison D.A., Rowan K., Aveyard P., Pavord I.D., Watkinson P.J. (2020). Risk of severe COVID-19 disease with ACE inhibitors and angiotensin receptor blockers: Cohort study including 8.3 million people. Heart.

[95] Gao C., Cai Y., Zhang K., Zhou L., Zhang Y., Zhang X., Li Q., Li W., Yang S., Zhao X. (2020). Association of hypertension and antihypertensive treatment with COVID-19 mortality: A retrospective observational study. Eur. Heart J..

[96] Angeli F., Verdecchia P., Balestrino A., Bruschi C., Ceriana P., Chiovato L., Dalla Vecchia L.A., Fanfulla F., La Rovere M.T., Perego F. (2022). Renin Angiotensin System Blockers and Risk of Mortality in Hypertensive Patients Hospitalized for COVID-19: An Italian Registry. J. Cardiovasc. Dev. Dis..

[97] Hasan S.S., Kow C.S., Hadi M.A., Zaidi S.T.R., Merchant H.A. (2020). Mortality and Disease Severity Among COVID-19 Patients Receiving Renin-Angiotensin System Inhibitors: A Systematic Review and Meta-analysis. Am. J. Cardiovasc. Drugs.

[98] Lopes R.D., Macedo A.V.S., de Barros E.S.P.G.M., Moll-Bernardes R.J., Dos Santos T.M., Mazza L., Feldman A., D’Andrea Saba Arruda G., de Albuquerque D.C., Camiletti A.S. (2021). Effect of Discontinuing vs Continuing Angiotensin-Converting Enzyme Inhibitors and Angiotensin II Receptor Blockers on Days Alive and Out of the Hospital in Patients Admitted With COVID-19: A Randomized Clinical Trial. JAMA.

[99] Kow C.S., Ming L.C., Hasan S.S. (2021). Renin-angiotensin system inhibitor use and the risk of mortality in hospitalized patients with COVID-19: A meta-analysis of randomized controlled trials. Hypertens. Res..

[100] Iacobucci G. (2021). COVID-19: People who take statins may be less likely to die, research suggests. BMJ.

[101] Hermida N., Balligand J.L. (2014). Low-density lipoprotein-cholesterol-induced endothelial dysfunction and oxidative stress: The role of statins. Antioxid. Redox Signal..

[102] Diaz-Arocutipa C., Melgar-Talavera B., Alvarado-Yarasca A., Saravia-Bartra M.M., Cazorla P., Belzusarri I., Hernandez A.V. (2021). Statins reduce mortality in patients with COVID-19: An updated meta-analysis of 147 824 patients. Int. J. Infect. Dis..

[103] Wu K.S., Lin P.C., Chen Y.S., Pan T.C., Tang P.L. (2021). The use of statins was associated with reduced COVID-19 mortality: A systematic review and meta-analysis. Ann. Med..

[104] Investigators I.-S. (2022). Atorvastatin versus placebo in patients with COVID-19 in intensive care: Randomized controlled trial. BMJ.

[105] Catanzaro M., Fagiani F., Racchi M., Corsini E., Govoni S., Lanni C. (2020). Immune response in COVID-19: Addressing a pharmacological challenge by targeting pathways triggered by SARS-CoV-2. Signal Transduct. Target. Ther..

[106] Gonzalez-Gay M.A., Castaneda S., Ancochea J. (2021). Biologic Therapy in COVID-19. Arch. Bronconeumol..

[107] Deng H., Tang T.X., Chen D., Tang L.S., Yang X.P., Tang Z.H. (2021). Endothelial Dysfunction and SARS-CoV-2 Infection: Association and Therapeutic Strategies. Pathogens.

[108] Alwazeer D., Liu F.F., Wu X.Y., LeBaron T.W. (2021). Combating Oxidative Stress and Inflammation in COVID-19 by Molecular Hydrogen Therapy: Mechanisms and Perspectives. Oxid. Med. Cell. Longev..

[109] Daiber A., Chlopicki S. (2020). Revisiting pharmacology of oxidative stress and endothelial dysfunction in cardiovascular disease: Evidence for redox-based therapies. Free Radic. Biol. Med..

[110] Carlstrom K.E., Ewing E., Granqvist M., Gyllenberg A., Aeinehband S., Enoksson S.L., Checa A., Badam T.V.S., Huang J., Gomez-Cabrero D. (2019). Therapeutic efficacy of dimethyl fumarate in relapsing-remitting multiple sclerosis associates with ROS pathway in monocytes. Nat. Commun..

[111] Timpani C.A., Rybalka E. (2020). Calming the (Cytokine) Storm: Dimethyl Fumarate as a Therapeutic Candidate for COVID-19. Pharmaceuticals.

[112] Ordonez A.A., Bullen C.K., Villabona-Rueda A.F., Thompson E.A., Turner M.L., Davis S.L., Komm O., Powell J.D., D’Alessio F.R., Yolken R.H. (2021). Sulforaphane exhibits in vitro and in vivo antiviral activity against pandemic SARS-CoV-2 and seasonal HCoV-OC43 coronaviruses. bioRxiv.

[113] Alam M.S., Czajkowsky D.M. (2021). SARS-CoV-2 infection and oxidative stress: Pathophysiological insight into thrombosis and therapeutic opportunities. Cytokine Growth Factor Rev..

[114] Schwalfenberg G.K. (2021). N-Acetylcysteine: A Review of Clinical Usefulness (an Old Drug with New Tricks). J. Nutr. Metab..

[115] Horowitz R.I., Freeman P.R., Bruzzese J. (2020). Efficacy of glutathione therapy in relieving dyspnea associated with COVID-19 pneumonia: A report of 2 cases. Respir. Med. Case Rep..

[116] Poe F.L., Corn J. (2020). N-Acetylcysteine: A potential therapeutic agent for SARS-CoV-2. Med. Hypotheses..

[117] Kim D.H., Meza C.A., Clarke H., Kim J.S., Hickner R.C. (2020). Vitamin D and Endothelial Function. Nutrients.

[118] Jain S.K., Micinski D., Parsanathan R. (2021). l-Cysteine Stimulates the Effect of Vitamin D on Inhibition of Oxidative Stress, IL-8, and MCP-1 Secretion in High Glucose Treated Monocytes. J. Am. Coll. Nutr..

[119] Gambardella J., Khondkar W., Morelli M.B., Wang X., Santulli G., Trimarco V. (2020). Arginine and Endothelial Function. Biomedicines.

[120] Fiorentino G., Coppola A., Izzo R., Annunziata A., Bernardo M., Lombardi A., Trimarco V., Santulli G., Trimarco B. (2021). Effects of adding L-arginine orally to standard therapy in patients with COVID-19: A randomized, double-blind, placebo-controlled, parallel-group trial. Results of the first interim analysis. EClinicalMedicine.

[121] Ambrosino P., Fuschillo S., Papa A., Di Minno M.N.D., Maniscalco M. (2020). Exergaming as a Supportive Tool for Home-Based Rehabilitation in the COVID-19 Pandemic Era. Games Health J..

[122] Tian F., Wang J., Xi X., He M., Zhao C., Feng F., Wang H., Sun W., Mao L., Hu X. (2021). Efficacy and safety of short-wave diathermy treatment for moderate COVID-19 patients: A prospective, double-blind, randomized controlled clinical study. Eur. J. Phys. Rehabil. Med..

[123] Parizad N., Goli R., Faraji N., Mam-Qaderi M., Mirzaee R., Gharebaghi N., Baghaie R., Feizipour H., Haghighi M.M. (2021). Effect of guided imagery on anxiety, muscle pain, and vital signs in patients with COVID-19: A randomized controlled trial. Complement. Ther. Clin. Pract..

[124] Divanoglou A., Samuelsson A.P.K., Sjodahl P.E.R., Andersson C., Levi P.R. (2021). Rehabilitation needs and mortality associated with the COVID-19 pandemic: A population-based study of all hospitalised and home-healthcare individuals in a Swedish healthcare region. EClinicalMedicine.

[125] Maniscalco M., Fuschillo S., Ambrosino P., Martucci M., Papa A., Matera M.G., Cazzola M. (2021). Preexisting cardiorespiratory comorbidity does not preclude the success of multidisciplinary rehabilitation in post-COVID-19 patients. Respir. Med..

[126] Sinoway L.I., Musch T.I., Minotti J.R., Zelis R. (1986). Enhanced maximal metabolic vasodilatation in the dominant forearms of tennis players. J. Appl. Physiol..

[127] Merlo C., Bernardi E., Bellotti F., Pomidori L., Cogo A. (2020). Supervised exercise training improves endothelial function in COPD patients: A method to reduce cardiovascular risk?. ERJ Open Res..

[128] Kitzman D.W., Brubaker P.H., Herrington D.M., Morgan T.M., Stewart K.P., Hundley W.G., Abdelhamed A., Haykowsky M.J. (2013). Effect of endurance exercise training on endothelial function and arterial stiffness in older patients with heart failure and preserved ejection fraction: A randomized, controlled, single-blind trial. J. Am. Coll. Cardiol..

[129] Dai R., Zhuo H., Chen Y., Zhang K., Dong Y., Chen C., Wang W. (2021). Mechanism of Isosorbide Dinitrate Combined with Exercise Training Rehabilitation to Mobilize Endothelial Progenitor Cells in Patients with Coronary Heart Disease. Bioengineered.

[130] Lanza G.A., Golino M., Villano A., Lanza O., Lamendola P., Fusco A., Leggio M. (2020). Cardiac Rehabilitation and Endothelial Function. J. Clin. Med..

[131] Montero D., Padilla J., Diaz-Canestro C., Muris D.M., Pyke K.E., Obert P., Walther G. (2014). Flow-mediated dilation in athletes: Influence of aging. Med. Sci. Sports Exerc..

[132] Ross M.D., Malone E., Florida-James G. (2016). Vascular Ageing and Exercise: Focus on Cellular Reparative Processes. Oxid. Med. Cell. Longev..

